# Identification of Two Missense Mutations in *DUOX1* (p.R1307Q) and *DUOXA1* (p.R56W) That Can Cause Congenital Hypothyroidism Through Impairing H_2_O_2_ Generation

**DOI:** 10.3389/fendo.2019.00526

**Published:** 2019-08-02

**Authors:** Shiguo Liu, Wenxiu Han, Yucui Zang, Hongwei Zang, Fang Wang, Pei Jiang, Hongwei Wei, Xiangju Liu, Yangang Wang, Xu Ma, Yinlin Ge

**Affiliations:** ^1^Medical Genetic Department, The Affiliated Hospital of Qingdao University, Qingdao, China; ^2^Prenatal Diagnosis Center, The Affiliated Hospital of Qingdao University, Qingdao, China; ^3^Department of Biochemistry and Molecular Biology, Medical School of Qingdao University, Qingdao, China; ^4^Institute of Clinical Pharmacy, Jining First People's Hospital, Jining Medical University, Jining, China; ^5^Department of Endocrinology and Metabolism, The Affiliated Hospital of Qingdao University, Qingdao, China; ^6^Center of Newborn Screening, Linyi Women and Children Hospital, Linyi, China; ^7^Prenatal Diagnosis Center, Taian Maternal and Child Health Hospital, Taian, China; ^8^Graduate School, Peking Union Medical College, Beijing, China; ^9^Center for Genetic Eugenics, National Research Institute for Family Planning, Beijing, China; ^10^World Health Organization Collaborating Center for Research in Human Reproduction, Beijing, China

**Keywords:** congenital hypothyroidism, *DUOX1*, *DUOXA1*, mutation, H_2_O_2_ generation

## Abstract

**Context:** The DUOX/DUOXA systems play a key role in H_2_O_2_ generation in thyroid cells, which is required for iodine organification and thyroid hormone synthesis. DUOX2/DUOXA2 defects can cause congenital hypothyroidism (CH), but it is unknown whether *DUOX1/DUOXA1* mutations can also cause CH.

**Objective:** We aimed to identify *DUOX1/DUOXA1* mutations and explore their role in the development of CH by investigating their functional impacts on H_2_O_2_ generation.

**Patients and Methods:** Forty-three children with CH with goiter were enrolled, in whom all exons and flanking intronic regions of *DUOX1/DUOXA1* were directly sequenced. We characterized the functional effects of identified mutations on the expression of *DUOX1* and *DUOXA1* and H_2_O_2_ generation.

**Results:** We identified a heterozygous *DUOX1* missense mutation (G > A base substitution at nucleotide 3920 in exon 31) that changed a highly conserved arginine to glutamine at residual 1307 (p.R1307Q) in patient 1. A heterozygous-missense mutation (c.166 C>T; p.R56W) was identified in *DUOXA1* in patient 2. Functional studies demonstrated that both p.R1307Q mutant or p.R56W mutant decreased the *DUOX1* expression at mRNA and protein levels, with a corresponding impairment in H_2_O_2_ generation (*P* < 0.01). The results also showed that intact DUOXA1 was required for full activity of DUOX1 and H_2_O_2_ generation.

**Conclusions:** We have identified two heterozygous missense mutations in *DUOX1* and *DUOXA1* in two patients that can cause CH through disrupting the coordination of DUOX1 and DUOXA1 in the generation of H_2_O_2_. This study for the first time demonstrates that the DUOX1/DUOXA1 system, if genetically defective, can cause CH.

## Introduction

Congenital hypothyroidism (CH) is the most common neonatal metabolic disorder which can result in abnormal growth and permanent mental retardation if treatment is delayed. Worldwide, CH affects 1 in 3,000–4,000 newborns and girls are more frequently affected than boys (female to male ratio is 2:1) (1). Most cases of CH are sporadic, and the occurrence of dyshormonogenesis caused by main enzyme defects in the thyroid hormone synthesis is generally inherited in an autosomal recessive manner and manifested as goitrous CH (2). Emerging evidence suggests that genetic defects in iodotyrosine deiodinase (*IYD*) (3), sodium-iodide symporter (*NIS*) (4), pendrin (*SLC26A4*) (5), thyroperoxidase (*TPO*) (6), thyroglobulin (*TG*) (7), dual oxidase 2 (*DUOX2*) (8), and dual oxidase maturation factor 2 (*DUOXA2*) (9) can cause goitrous CH, including transient or permanent types.

Hydrogen peroxide (H_2_O_2_) plays a key role not only in iodine organification but also in tyrosine iodination and is thus critical to the synthesis of thyroid hormone (10). As members of the NADPH oxidase family, dual oxidases (DUOX1 and DUOX2) are supposed to be the sources of reactive oxygen species which can transfer one electron to iodine in iodine organification (11). Both DUOX1 and DUOX2 play a critical role in the production of H_2_O_2_ in the thyroid gland, which is the limiting factor in thyroglobulin iodination and thyroxine synthesis (12, 13). Interestingly, initial molecular studies demonstrated that no peroxide production could be detected even though DUOX family contains all characteristics consistent with NADPH oxidases (14). Subsequent studies demonstrated that DUOX enzymes were inactive due to its immature form inside the endoplasmic reticulum (ER) and only if the formation of heterodimeric complex consisting of DUOX and an additional partner, dual oxidase maturation factor (DUOXA), was accomplished, could the DUOX enzymatic activity be expressed (14). Thus, DUOXA is required for DUOX to form functional complexes, which is essential for ER-to-Golgi transition, maturation and translocation to the plasma membrane. Only DUOX1/DUOXA1 and DUOX2/DUOXA2 complexes appear to be fully functional *in vitro*, whereas the DUOX2/DUOXA1 and DUOX1/DUOXA2 complexes appear to be unstable and tend to dissociate at the cell surface (9, 15).

Although increasing studies have demonstrated that *DUOX2/DUOXA2* mutations are associated with CH, it is unclear whether *DUOX1/DUOXA1* defects can similarly cause CH. Hoste et al. (16) reported some patients with complete inactivation of DUOX2 who manifested with transient CH early in life, suggesting that DUOX2 defects can be compensated fully by DUOX1 after the neonatal period is completed. However, no *DUOX1* mutations and related genotype-phenotype manifestations have been identified in goitrous CH patients. Moreover, the identification of novel *DUOXA2* missense mutation in a transient CH patient with a large deletion comprising *DUOX2, DUOXA2*, and *DUOXA1* (17) suggests that *DUOXA1* mutation may lead to CH as *DUOXA2* mutation does. Therefore, it is necessary to investigate the relationship between *DUOX1/DUOXA1* defects and the development of CH. In the present study, we screened goitrous CH patients for *DUOX1* and *DUOXA1* mutations in a Chinese population, investigated the corresponding genotype-phenotype correlation, and characterized the functional effects of the identified mutations on *DUOX1* and *DUOXA1* at the molecular level.

## Materials and Methods

### Patients

Forty-three (28 boys and 15 girls, male-female ratio 1.87:1.0, mean age 0.06 ± 0.03 years) cases of CH with goiter were identified in the neonatal screening at neonatal screening centers in Shandong province, China. The neonati were given full breast feeding 72 h after birth. Their heel blood was dropped in a dry blood spot on a filter paper to examine thyroid stimulating hormone (TSH) concentration for screening. Thyroxine (T4), triiodothyronine (T3), TSH, free thyroxine (FT4), and free triiodothyronine (FT3) in the serum were formally measured when the subjects had elevated TSH (≥20 μIU/ml) levels on the screening test. The subjects were diagnosed with CH if they had abnormally elevated TSH concentration (normal range 0.27–4.2 μIU/ml) and low FT4 concentration (normal range 12–22 pmol/L). This biochemical diagnosis of CH was followed by a 99mTc thyroid scan or thyroid ultrasound examination in the patients with eutopic thyroid gland. All the subjects from 43 unrelated families were free from other congenital diseases. Our study was approved by the medical ethics committee of the Affiliated Hospital of Qingdao University with informed consent of patient families.

### DNA Analysis of *DUOX1* and *DUOXA1*

Genomic DNA was isolated from peripheral blood leucocytes of patients and stored at −20°C up to the time of analysis. The complete sequences of *DUOX1* (35 exons) and *DUOXA1* (11 exons), including splice and flanking intronic regions, were amplified by polymerase chain reaction (PCR). The primer sequences of all exons in *DUOX1* and *DUOXA1* are listed in Tables 1, 2, respectively. Mutations in *DUOX2* (10), *DUOXA2* (9), *TPO, TG*, and *NIS* (18) for all subjects had been excluded in our previous study in these patients. The PCR reaction system (25 μL) consisted of 100 ng of genomic DNA, 1 U of Taq DNA polymerase, 500 μM dNTPs, 0.5 μM each primer and 1×reaction buffer (20 mM Tris-HCl, pH 8.0, 3 mM MgCl_2_). Samples were pre-denatured at 95°C for 5 min, followed by PCR reaction for 35 cycles of 30 s at 95°C, 30 s at 55–61°C, and 30 s at 72°C, with a final extension at 72°C for 7 min. Purified PCR products were analyzed on 2% agarose gel electrophoresis and mutations were identified by analyzing the nucleotide sequence (NC_000015.10) after ABI 3730XL (Applied Biosystems) sequencing.

**Table 1:** The primer sequences of all exons of *DUOX1* gene.

**Primer name**	**Forward sequence 5^′^ → 3^′^**	**Reverse sequence 5^′^ → 3^′^**	**Product length (bp)**
1	TCGGCACCGACGGAACAT	TTGATTGGGCAGAGGACAGG	492
2	ATTAAACCTCCTTCTCACA	GATTCGGCAAATACTTCA	369
3	TTTGCTACCTACTGTGACC	ATCCATCTATTTCTTCAACA	396
4	CCCTTCCCTCCATTCTCA	TGGTACACGCCATCTGCA	356
5	GCCATCCATTTCCAAGGT	CATCCTCTGACCCCTCTTC	355
6 + 7	TCAGGGGAGGGAAGGAAACT	CCAGGAATGCGAGGAACCA	518
7 + 8	TATGGTTCCTCGCATTCCTGG	GGTTTCTGGGTGGCGGTTGT	543
9 + 10	TGCTCCTGTTTGAGTTGCT	CTCCTGAGGGCAGATTTT	489
11	GGACCTTCCAAAAGTCAGACCCTC	AGCCTTACTGACCACTTTCACCATG	381
12	GGAAACATCCCATTAGACAC	ACAGCCTCAGACTCACCC	784
13	AAGCACCCAAGCCTACAA	TTTCACTGCCTATCTTCCAT	618
14–1	AGTGGGATTAAGCTGGTC	CTAAGGCAAAGAGGTGGC	459
14–2 + 15	GCCACCTCTTTGCCTTAG	GATTGACGTTCAGCCACAT	675
16	GGAGGTTGGGATTCATTT	TTTGCGTGGTTTGTTGTC	395
17	GTGGTATTGCCAGGTAAGG	CCAGGGATTCGTAAGTCATAG	471
18	CTTCCTCCCAATGTACCTCT	GACTCCTGCACAGTAACTATCA	378
19	CCCCTTTCCTCTTCTGTAA	CTCTGGGCTTTGACTTCC	422
20	GCTGCCTTTGCCTTACTT	CTATCATTCCCTCCTCCCT	566
21	ATGGCTACCTGTCCTTCCG	CCTTGGCCCTTCTGCTCT	485
22	AACAACTGCCTGTCCAAG	CCATTTCCTACCCTATCCT	479
23–1	CTCAGCCTAGACAGGAACA	CTCAAACGCACAGCACTC	451
23–2	TTTTCCCTGCCCACCTAT	ATGCCTTTACCCTTCACAA	504
24	TCCTCAGCAGAATGGGTT	TTAGAACAGTCATCAGTGGG	417
25	TTTCTTTCTCGGAAGCAGTG	CAAAGGCGTAGTCTGTGGAG	544
26–1	CCTGGGTAGGGTAAGTGGA	TGGAAAGTGCGGGAAGAG	578
26–2	TCTGCTGGCACTTACCTTT	CACTCATTCAGCCCAAGAT	733
27	ATGAGTGAGCACCCACCCTG	CCTCCTGGTTTCAAGCCATTCT	497
28	CCTCTCAAGGTGTCTCTTTGCTGTC	AGGAGGGCTGGAATGCAGAGTAG	274
29	GGGACATTCTTACTCCAACT	CTGTACTATCATCTCCACCTTC	398
30	ATGGAGTTGGGAATGGTG	TGGGATTACAGGCGTGAG	495
31	CTTCTCACCCACCATCCC	CTCCCACTAACACTGACACCTC	579
32	AGAACAAAGGCAGAGGAGA	ACAGATGTGCCAGATACCC	519
33	GCCCATACACTCCATCTCC	GAAGTGCCGCTCACAGAT	421
34	TTGTTCCACCCTTCCCTA	ACCCACCTATCCTTTCCA	308
35–1	CAGGGATGTTGCTATGTTG	TATGGGAGTAGGGAGGGT	665
35–2	CTCCCAACCTTGTTCCAG	TGGGTTAGTGGCTTCCTC	628

*DUOX1, dual oxidase 1*.

**Table 2:** The primer sequences of all exons of *DUOXA1* gene.

**Primer name**	**Forward sequence (5^′^ → 3^′^)**	**Reverse sequence (5^′^ → 3^′^)**	**Product length (bp)**
1	AGCCCTCCCAAATCTGACCT	GGCACCGACGGAACATCTC	316
2	TCCGCCTTCACAAGTCCC	AGCTCCAGCGCAAACCTAG	312
3	TCTGAGAAGTTTGGGAGTGAC	TCTGGATGAAAGCAGGAAGT	405
4	GCAGTGGAACGGTGGTAA	CTCCTGGGCTCAAGCAAT	590
5	TAGCAGAGTCTGATGATGCACAAA	CCACCAGCGCTCAATAGTGA	500
6	AAAAAATAGCTGGGCATCATGGT	GGAAGCCACCCTGAAGCAA	500
7	TGGTGTGGTCAAAGAGCTATAGGAT	GGGTAGAAACCCTGTTCCTGAA	500
8	GGGTAGAAACCCTGTTCCTGAA	GGGAGAGAAAATCAGGAGATAAGAGA	501
9–1	TAGGCTTCCCTTAGAGTTGTTCTGA	TTGGCCTTATCATGGCAACAG	600
9–2	CCTCAGGGTGGCTGTTACCA	TTCCCACCTGGCTTCTTGTG	600
9–3	TTCCCACCTGGCTTCTTGTG	CCAGACTTAAAATGTATCACCACTAACC	677
9–4	CCAGACTTAAAATGTATCACCACTAACC	TGCACTTTCCAGTTTACAGAATGAA	642
10	TGCCTCATCACTGCCACCTA	CGCTCTTCGCACCCTTCT	563
11	GGTCGCCGAGGATAAGAG	GCTAAGGGTGGAGACAGGAT	520

*DUOXA1, dual oxidase maturation factor 1*.

### cDNA and Construction of *DUOX1/DUOXA1* Expression Vector

After extraction of RNA from human thyroid tissue, cDNA was synthesized by the primer oligo (dT) through the reverse transcription reaction. The consensus coding sequence of *DUOX1* (4,656 bp) was amplified from cDNA (forward primer: 5′-ATGGGCTTCTGCCTGGCTCTA-3′, reverse primer: 5′-CTAGAAGTTCTCATAATGGTG-3′) and was cloned into pEASY-Blunt M2 expression vector. Similarly, the consensus coding sequence of *DUOXA1* (1,452 bp) was amplified from cDNA (forward primer: 5′-ATGGCTACTTTGGGACACACA-3′, reverse primer: 5′-TCAGATTAGAGGTGTGTGGCG-3′) and was cloned into pEASY-Blunt M2 expression vector. ***DUOX1* p.R1307Q** mutant was introduced by site-directed mutagenesis (Fast Mutagenesis System) into the expression vector using the mutagenic forward primer 5′-GAGCGGCCAGTGGGTCCAAATCGCTTGTC-3′ and the mutagenic reverse primer 5′-TGGACCCACTGGCCGCTCTTGTACTCGAA-3′. The expression vector of mutant ***DUOXA1* p.R56W** was constructed on the basis of wild type expression vector using the mutagenic forward primer 5′-GGCTGTTCTGGCTGCTTTGGGTGGTGACC-3′ and the mutagenic reverse primer 5′-AAAGCAGCCAGAACAGCCTCGTCTTTCCC-3′ in the same way. All the constructed expression vectors were confirmed by DNA sequencing.

### Cell Culture and Transient Cell Transfection

HeLa cells were grown in six-well plates in 1640 culture medium supplemented with 10% fetal calf serum in a humidified condition with 5% CO_2_ at 37°C. HeLa cells were divided into eight groups according to the transfected expression vector type before transfection, including DUOX1 (containing *DUOX1* wild type [WT] expression vector only), DUOXA1 (containing *DUOXA1* WT expression vector only), DUOX1 and DUOXA1 (containing both *DUOX1* WT and *DUOXA1* WT expression vector), p.R1307Q and DUOXA1 (containing *DUOX1* p.R1307Q mutant expression vector and *DUOXA1* WT expression vector), DUOX1 and p.R56W (containing *DUOX1* WT expression vector and *DUOXA1* p.R56W mutant expression vector), p.R1307Q and p.R56W (containing both *DUOX1* p.R1307Q and *DUOXA1* p.R56W mutant expression vectors), pEASY-Blunt M2 (containing an empty vector), and parental non-transfected cells (containing no expression vector). When HeLa cells reached 70–80% confluence, they were transfected with 4 μg of plasmid DNA (WT, mutant, and control) in six-well plates using TransIn EL Transfection Reagent (TransGen, Beijing, China). To ensure quantitative consistency of plasmids, empty plasmid was used as a reference in individual transfected groups and the DUOX1/DUOXA1 ratio was 7:1. The culture medium was replaced at 6 h after transfection and cells were harvested after 48 h.

### mRNA Expression of *DUOX1* and *DUOXA1*

HeLa cells cultured on six-well plates were suspended in Trizol reagent (Invitrogen, Carlsbad, USA) and total RNA was purified 48 h after transfection. Reverse transcription was performed using a Prime Script (™) RT Enzyme Mix I (TaKaRa, Shiga, Japan) on 1 μg of total RNA in a reaction volume of 20 μL. One-fifth of the cDNA product was used for real time PCR amplification using TIANGEN Taq DNA polymerase (TIANGEN, Beijing, China) and the amplified PCR products were detected according to the fluorescence intensity of SYBR Green I. For specific amplification of human *DUOX1* cDNA, forward primer was 5′-TCTGGGACTGCTCTGGTTC-3′ and reverse primer 5′-CGATGTTCTGGTAGGTGGC-3′; for human *DUOXA1*, forward primer was 5′-AAAGGCTCTGGAGAAGGG-3′ and reverse primer 5′-GCATGGCTGAGGTGTAGTG-3′; for *GAPDH* (loading control), forward primer was 5′-AGAAGGCTGGGGCTCATTTG-3′ and reverse primer 5′-AGGGGCCATCCACAGTCTTC-3′. Samples were heated to 95°C for 15 min, followed by 40 cycles of 10 s at 95°C, 32 s with annealing/elongation at 63°C. The amplified level of the target gene was normalized against that of *GAPDH*.

### Western Blot Analysis of DUOX1 Expression

After transfection for 48 h, monolayer cells were suspended in cell lysis solution containing protease inhibitors (Phenyl methane sulfonyl fluoride, PMSF) on ice for 30 min after washing with physiological saline. Total protein was collected from the supernatant of cell lysates after centrifugation for 20 min at 4°C. After measurement of total protein concentration using a BCA Protein Quantitative Kit (TransGen, Beijing, China), protein samples (30 μg) were denatured by heating water at 95°C for 10 min in the loading buffer (TransGen, Beijing, China), subjected to SDS-PAGE using a 8% acrylamide mini-gel, and transferred to a PVDF membrane. The PVDF membrane was blocked for 2 h at room temperature with 5% nonfat dry milk in PBS (pH7.4) and 0.1% Tween 20 and was then incubated overnight at 4°C with primary antibodies against DUOX1 (Bioss, Beijing, China) at a 1:250 dilution and against beta-Actin (Loading Control) at a 1:2,000 dilution in PBS-Tween 20 containing 5% nonfat dry milk. Membranes were washed, incubated for 1 h with peroxidase-labeled secondary antibody anti-rabbit, washed again, and visualized with enhanced chemiluminescence on CL-XPosure films.

### Measurement of H_2_O_2_ Generation

Extracellular H_2_O_2_ generation was quantified by the Amplex red/horseradish peroxidase assay (Molecular Probes, Invitrogen), which detected the accumulation of a fluorescent oxidized product. Amplex Red reagent (10-acetyl-3,7-dihydroxyphenoxazine, 50 μM) and horseradish (0.1 U/ml) peroxidase (HRP) was added into each well after transfection for 48 h and the six-well plate was placed in an incubator with dark environment at 37°C for 1 h. The fluorescence intensity was measured by Synergy (™) multi-functional enzyme marking instrument using excitation at 535 nm and emission at 595 nm. H_2_O_2_ release of experiment groups and control groups were quantified using standard calibration curves (as shown in Supplementary Figure 1) which could change the fluorescence intensity into absolute nanomoles of H_2_O_2_.

### Protein Synthesis Suppression Assay

Cycloheximide (CHX) at 100 μg/ml was added to block the synthesis of intracellular protein and total protein was extracted after cells were disassociated in four different time periods within 24 h. The DUOX1 protein expression was detected by Western blot.

### Statistical Analysis

Statistics analysis of variance (ANOVA) was used for data presented as mean ± standard deviation (SD) using the software of SPSS 17.0, followed by LSD (least significant difference) multiple comparison test. A *P* < 0.05 was considered to be statistically significant.

## Results

### Mutation Screening and Genotype-Phenotype Analysis

**Figure 1:**
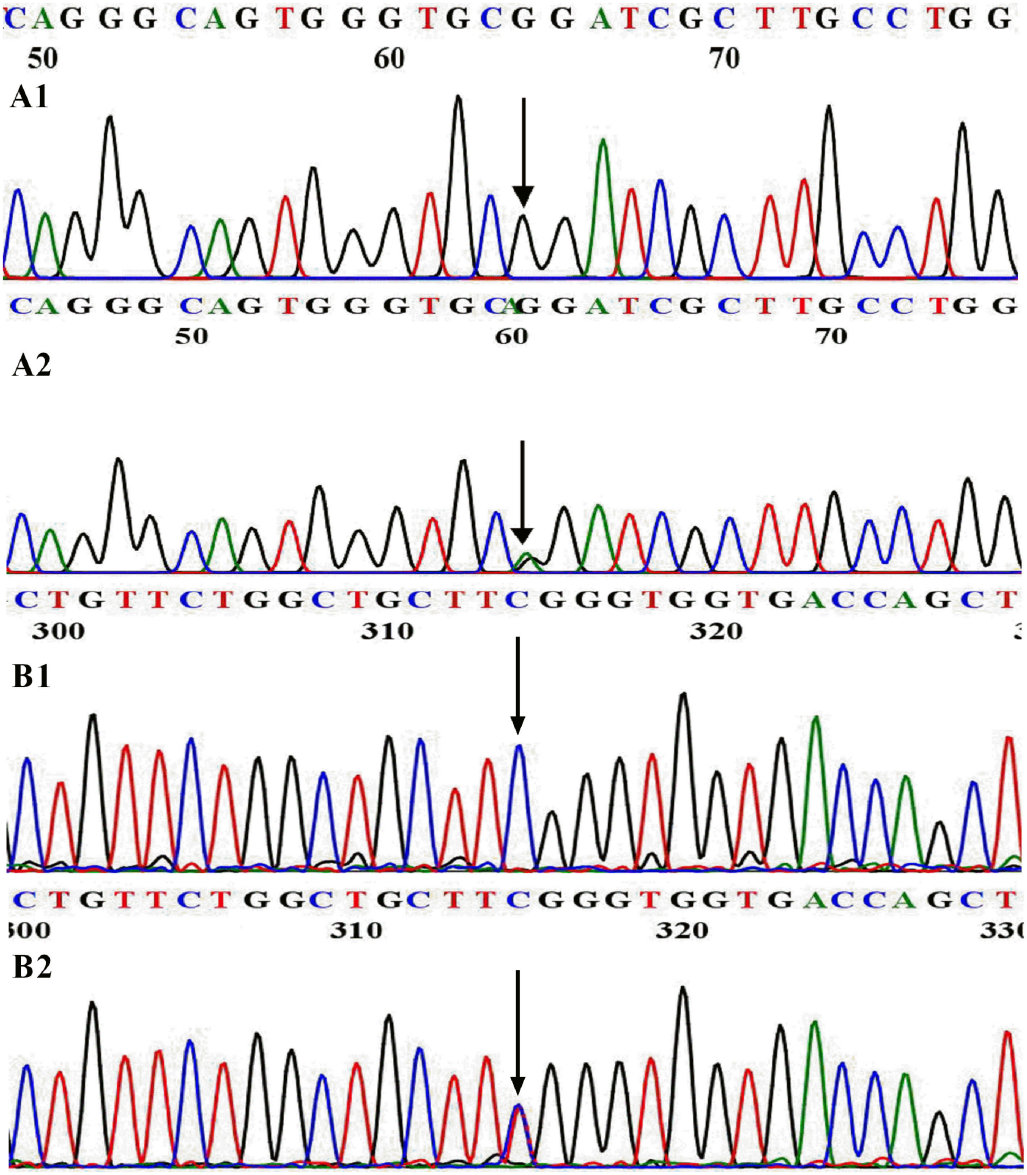
WT and mutational sequences of exon 31 in *DUOX1* (A) and exon 6 in *DUOXA1* (B). **(A1)** Arrowhead indicates homozygous G at nucleotide 3920 in normal individuals; **(A2)** Arrowhead indicates the heterozygous A and G at nucleotide 3920 in patient 1. **(B1)** Arrowhead indicates homozygous C at nucleotide 166 in normal individuals; **(B2)** Arrowhead indicates the heterozygous C and T at nucleotide 166 in patient 2.

**Figure 2:**
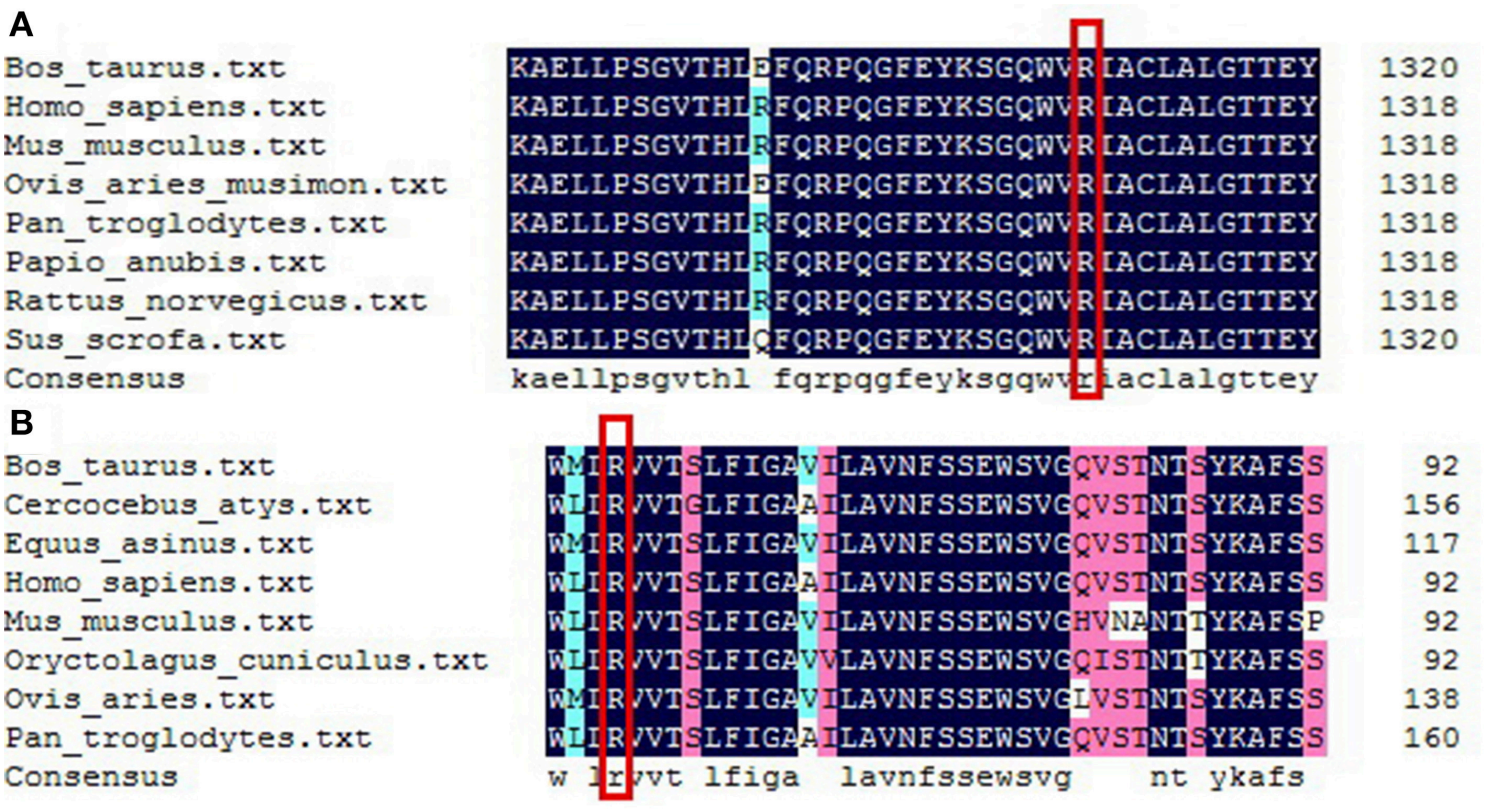
Sequence alignment analysis of DUOX1 **(A)** and DUOXA1 **(B)** in different species. **(A)** Red rectangle indicates arginine at amino acid 1307of DUOX1 located in a conserved sequence of the DUOX1 protein. **(B)** Red rectangle indicates arginine at amino acid 56 of DUOXA1 located in a conserved sequence of the DUOXA1 protein.

A heterozygous missense mutation (c.3920G>A, present in Genome Aggregation Database, Figure 1A) was identified in the *DUOX1* gene in patient 1 (P1), causing an arginine-to-glutamine substitution at amino acid 1307 of DUOX1, which was located in a conserved sequence of the protein according to the multiple sequence alignment analysis (Figure 2A). This mutation was not found in 100 healthy control Chinese subjects. The relationship was not clear between *DUOX1* mutation segregation and phenotype within the family because of the lack of relevant data. P1 was a female infant with birth weight of 2,500 g. She was born at full-term gestation by cesarean delivery and had no family history of thyroid disease. She was called back to hospital at 36 days (body weight 3,800 g, height 47 cm) after birth for further evaluation because of her high TSH concentration (>100 μIU/ml) on the filter paper neonatal screening test. Her thyroid gland was enlarged on the 99mTc thyroid scan and the perchlorate discharge test was positive. Levothyroxine (L-T4) replacement therapy was started immediately with the initial dose of 7.7 μg/kg. To maintain normal serum TSH and FT4 levels, replacement therapy dose was adjusted based on her clinical and hormonal evaluations during the subsequent follow-up. TSH, FT3, and FT4 levels remained normal following a temporary withdrawal of L-T4 therapy for 4 weeks when she was 2 years old. Her final diagnosis was therefore transient CH. The patient is currently 10.5 years old with normal physique and intelligence.

Patient 2 (P2) carried a *DUOXA1* heterozygous missense mutation p.R56W (a C>T transversion at nucleotide 166 in exon 6, present in Genome Aggregation Database, Figure 1B) that changes the highly conserved arginine at amino acid 56 of DUOXA1 protein (Figure 2B) to tryptophan and resulted in severe congenital hypothyroidism, which was not identified in 100 healthy control Chinese subjects. P2 was a male subject (birth weight 2,750 g) with a p.R56W mutation, born by normal delivery at full-term gestation. He was called back to hospital after neonatal screening at 35 days from birth for a high level of TSH (>100 μIU/ml) on the filter paper screening test; he was subsequently diagnosed with CH with a low serum FT4 level (4.7 pmol/L). A 99mTc thyroid scan indicated that P2 had eutopic thyroid gland with mild diffuse goiter. L-T4 replacement therapy was started immediately at an initial dose of 8.4 μg/kg per day. To maintain normal serum TSH, FT3, and FT4 levels, the dose was adjusted according to clinical and hormonal evaluations. The patient continued to have hypothyroidism requiring thyroid hormone therapy at 2.5 years; this patient was therefore diagnosed with permanent CH. The patient is currently 9.5 years old and his physical and mental developments are normal. She currently receives L-T4 with a daily dose of 5.9 μg/kg.

### Effects of Mutations on the Expression of *DUOX1/DUOXA1*

**Figure 3:**
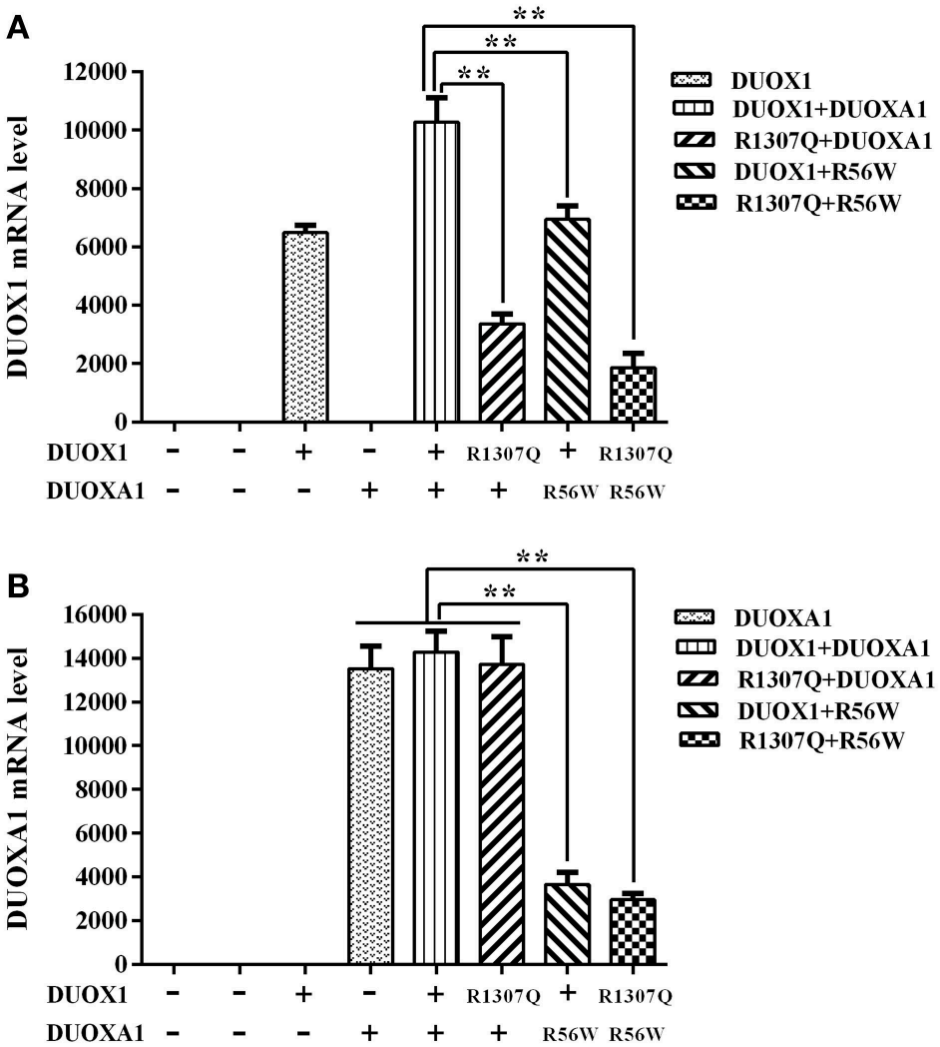
Relative mRNA expression levels under the various indicated vector transfections. **(A)** *DUOX1* mRNA expression. The *DUOX1* mRNA level was highest in the group with both wild-type *DUOX1* and *DUOXA1* expression vectors, while the group with p.R1307Q and p.R56W combination had the least mRNA expression of the *DUOX1* gene compared with other experimental groups except for the group with *DUOXA1* only. **(B)** *DUOXA1* mRNA expression. Groups with p.R56W mutant were far lower than other experimental groups with *DUOXA1*. Each column represents the mean ± SD of three independent experiments performed in triplicate. ^**^P < 0.01.

The relative expression of *DUOX1* mRNA decreased with mutant constructs in comparison with both wild type constructs (*P* < 0.01), and the group with p.R1307Q and p.R56W combination (p.R1307Q + p.R56W) had the least expression of *DUOX1* mRNA compared with other groups (*P* < 0.01). With respect to the relative expression of *DUOXA1* mRNA, groups with p.R56W mutant (DUOX1 + p.R56W or p.R1307Q + p.R56W) were far lower than other groups except for the group with *DUOX1* only (*P* < 0.01) (Figure 3).

**Figure 4:**
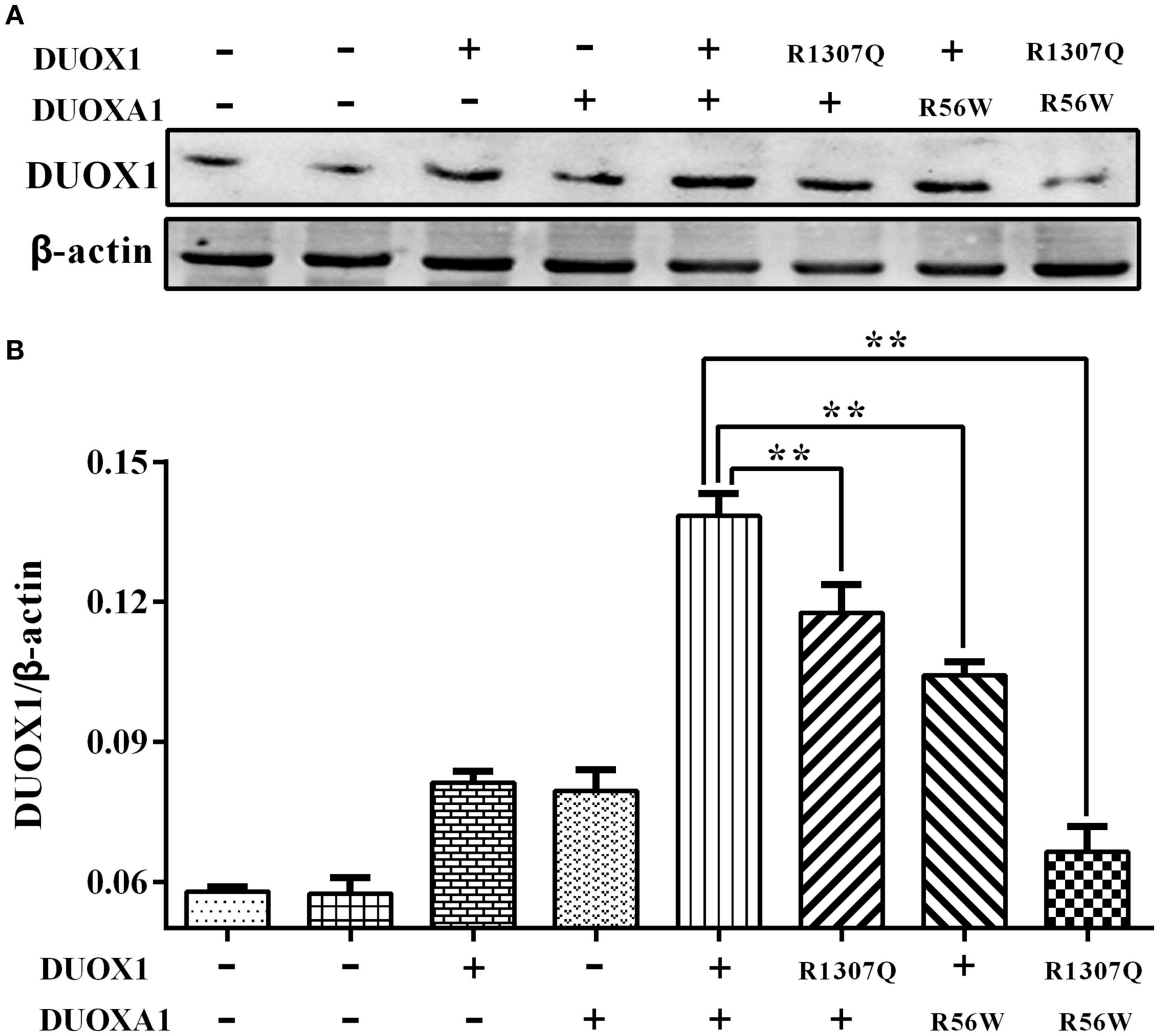
Western blot analysis of the DUOX1 protein expression under various indicated vector transfections. **(A)** Representative presentation of the gel results. The DUOX1 protein level was highest in the group with both wild-type DUOX1 and DUOXA1 expression vectors, while the group transfected with both p.R1307Q mutant and p.R56W mutant had the least expression level. **(B)** Densitometric measurement of DUOX1 protein expression. The results are presented as the ratio of DUOX1/β-actin, corresponding to **(A)**. Primary antibodies against DUOX1 was used at a 1:250 dilution and Anti-beta-actin, as loading control, was used at a 1:2,000 dilution. Each column represents the mean ± SD of three independent experiments performed in triplicate. ^**^P < 0.01.

Consistent with the results of mRNA expression, both p.R1307Q and p.R56W were associated with decreased expression of DUOX1 protein compared with the wild type protein on the western blot analysis (*P* < 0.01). In particular, the expression of DUOX1 protein in HeLa cells transfected with both p.R1307Q and p.R56W in combination decreased most significantly (*P* < 0.01) (Figure 4). The size of the bands of DUOX1 protein is presented in Supplementary Figure 2.

### H_2_O_2_ Production Assay

**Figure 5:**
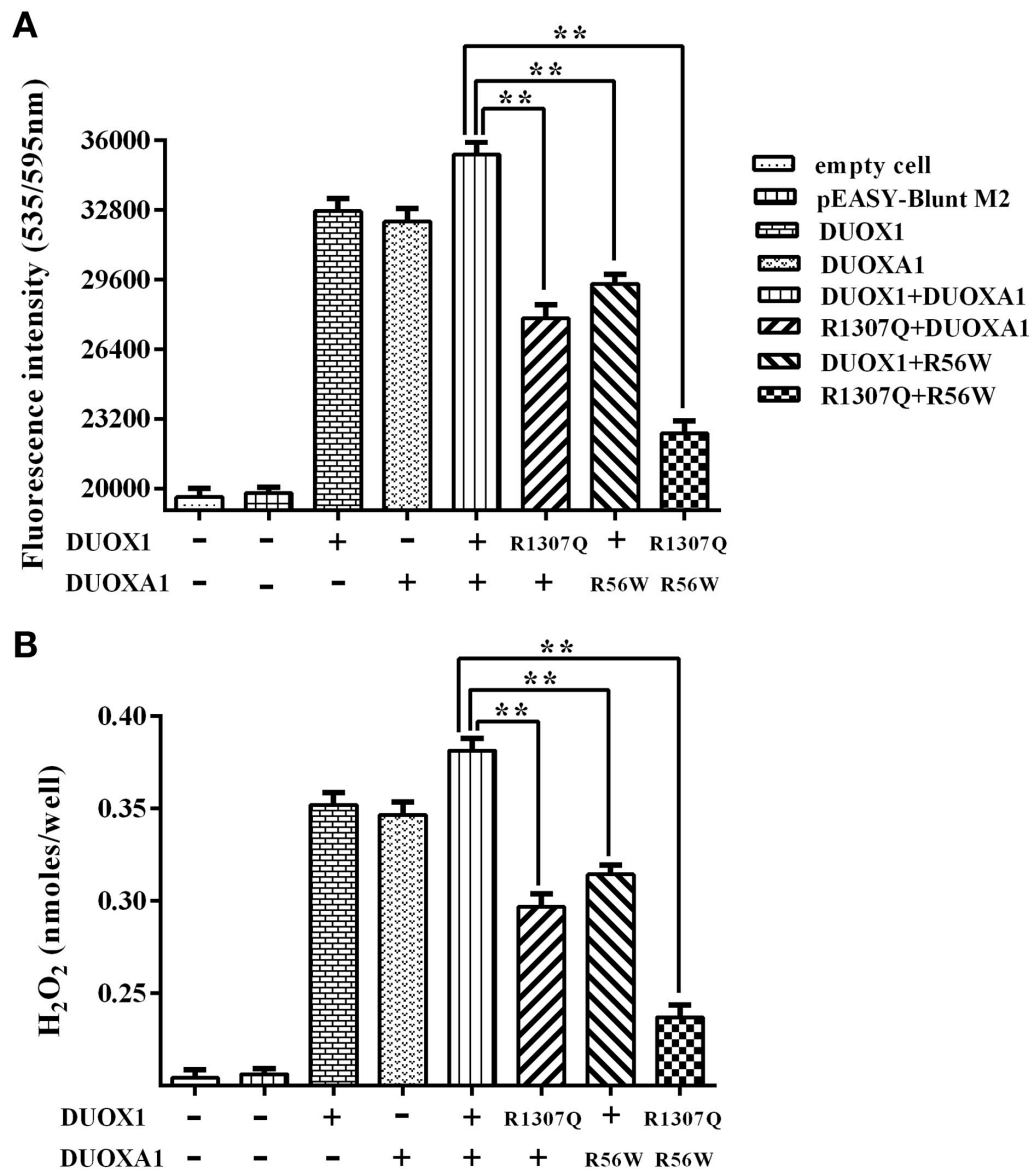
H_2_O_2_ generation in cells transfected with the indicated expression vectors. **(A)** Measurement of fluorescence intensity (535/595 nm) under various indicated vector transfections. The group transfected with both wild-type DUOX1 and DUOXA1 had the maximum fluorescence intensity. **(B)** Presentation of the data in nanomoles of H_2_O_2_. The results were transformed from fluorescence intensity using a calibration curve. Each column represents the mean ± SD of three independent experiments performed in triplicate. ^**^P < 0.01.

As shown in Figure 5, transfection with wild-type *DUOX1* or *DUOXA1* each alone significantly increased H_2_O_2_ production compared with empty vector transfection. Transfection with mutant p.R1307Q or p.R56W alone resulted in reduced H_2_O_2_ production compared with their corresponding wild-type counterparts, respectively (*P* < 0.01). The group transfected with both wild-type *DUOX1* and *DUOXA1* in combination (*DUOX1* or *DUOXA1*) produced the most amount of H_2_O_2_ (*P* < 0.01) while, in contrast, the group with transfection of both mutants p.R1307Q and p.R56W in combination (p.R1307Q + p.R56W) impaired the H_2_O_2_-generating system to the maximum extent (P<0.01). These patterns were consistent with the Western blot analysis results above (Figure 4). Thus, these *DUOX1* and *DUOXA1* mutants were able to substantially inhibit the functional activities of the H_2_O_2_-generating system, demonstrating the functional consequences of the two mutations.

### Effects of CHX on DUOX1 Expression

**Figure 6:**
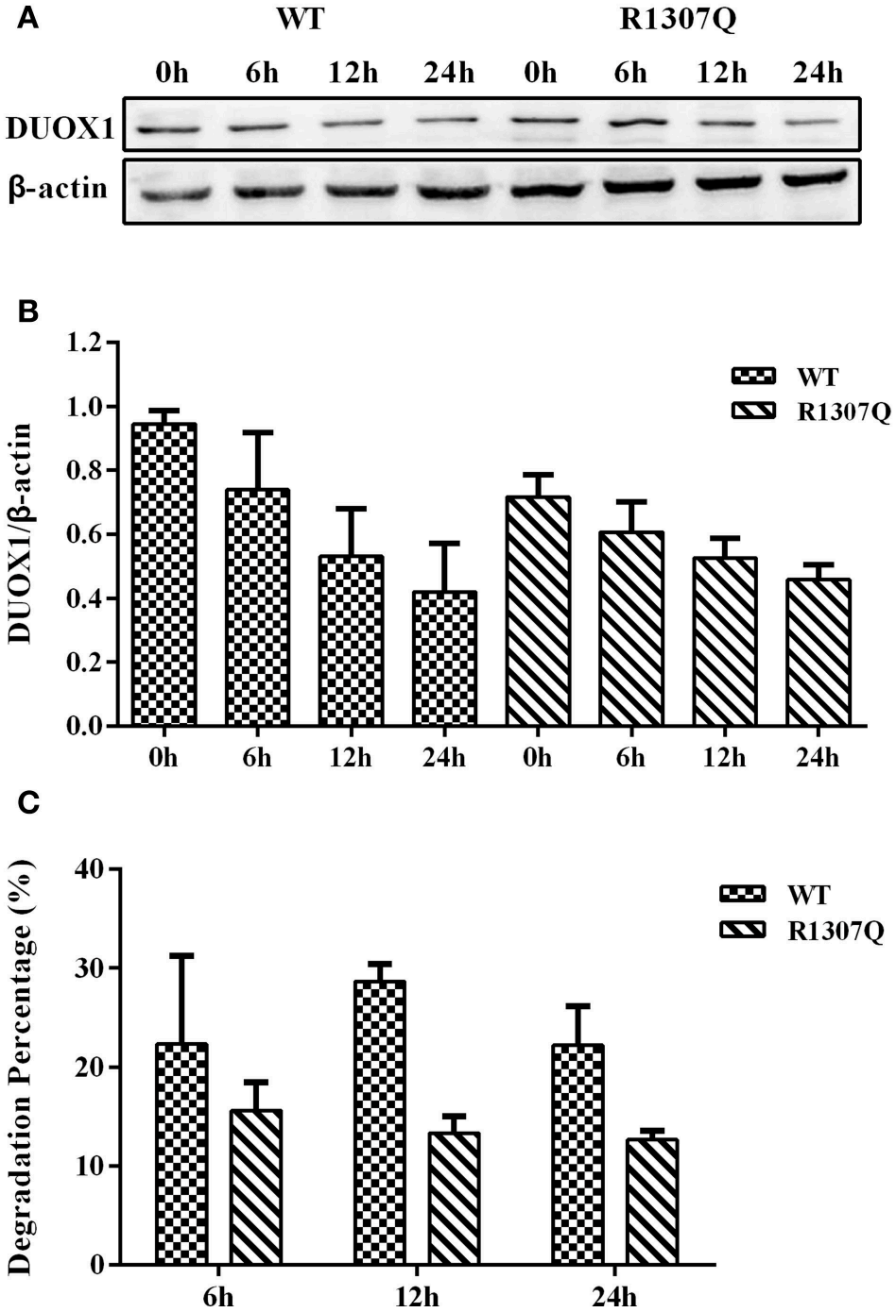
Cycloheximide (CHX) chase study. **(A)** DUOX1 protein expression was examined at different time periods in cells transfected with WT or p.R1307Q expression vectors. **(B)** DUOX1 expression was quantified densitometrically as the ratio of DUOX1/β-actin. **(C)** Degradation percentage of DUOX1 protein in cells transfected with WT or p.R1307Q expression vectors. Each column represents the mean ± SD of three independent experiments performed in triplicate.

DUOX1 protein synthesis with transfection of all expression vectors for 24 h was blocked with the addition of CHX. Compared with the quantity of DUOX1 protein at the beginning of adding CHX (0 h), the degradation rate of mutant DUOX1 protein had no significant difference compared with wild type DUOX1 protein at 6, 12, and 24 h after transfection (*P* > 0.05; Figure 6).

## Discussion/Conclusion

A crucial step in thyroid hormone biosynthesis is iodine organification, which can only be successfully accomplished in the presence of H_2_O_2_. The NADPH oxidases DUOX1 and DUOX2, also known as thyroid oxidases ThOX1 and ThOX2, were originally identified in the thyroid tissue thanks to the cloning of the human cDNA encoding NADPH oxidase (12, 13). The two corresponding genes (*DUOX1* and *DUOX2*) are located on chromosome 15q15.3 and encode 1,551 and 1,548 amino acids in length, respectively. As seventh-pass-membrane proteins, DUOX proteins are composed of the extracellular peroxidase-like domain, transmembrane domain, two EF-hand domains, one FAD-binding site, and four nicotinamide adenine dinucleotide phosphate (NADPH)-binding sites (13, 19–21). As a candidate thyroid H_2_O_2_ generator, the activity of DUOX is likely to adapt to the feedback of the local H_2_O_2_ concentrations (22, 23). Recent studies showed that the activation of DUOX-based H_2_O_2_ generator relies on the formation of a heterodimer complex consisting of DUOX and a specific maturation factor (DUOXA1 and DUOXA2) (24). *DUOXA1* and *DUOXA2* were first cloned and named in Chicago in 2006 (25). They are located on chromosome 15 and consist of 6 (*DUOXA2*) and 11 (*DUOXA1*) exons, encoding transmembrane proteins of 320 (DUOXA2) and 483(DUOXA1) amino acids, respectively. Other than the thyroid tissue, DUOXA2 can be detected in the epithelium of the digestive tract at low levels (26).

The finding of the association of DUOX2/DUOXA2 system with CH led to the identification of their predominant role in thyroid hormone synthesis. It is well-known that *DUOX2/DUOXA2* mutations can cause CH directly, while it is controversial whether the DUOX1/DUOXA1 system can also play a role in CH. It was previously believed that DUOX1 had no relationship with CH due to the fact that DUOX2 deficiency could result in severe hypothyroidism, indicating that DUOX1 could not compensate the deficiency and was therefore not important in thyroid hormone synthesis (27). In addition, *DUOX1* knockout mice had normal growth and serum thyroxin levels, supporting this belief. Nevertheless, the above hypothesis was questioned by later studies which demonstrated that DUOX1 participated partially in thyroid hormone synthesis when complete loss of DUOX2 activity occurred (28). Moreover, the clinical identification of a patient with Y246X homozygous nonsense mutation in *DUOXA2* also suggested such a function of DUOXA1 under the circumstances of DUOXA2 inactivation (9). Therefore, it is interesting and important to explore the role of the DUOX1/DUOXA1 system in CH.

In the present study, we found that P1 with CH with goiter carried a heterozygous missense mutation (c.3920G>A, p.R1307Q) in *DUOX1*. The highly conserved amino acid arginine coded by the wild type codon where the mutation occurred is localized in the intracellular domain of C terminal near the FAD binding domain. Clinical data indicated that this patient indeed had a partial defect of H_2_O_2_ generation in the DUOXA1 reconstitution assay and manifested biochemically as a transient form of CH in the neonatal and infantile period. Subsequent studies showed that the requirements of thyroid hormone synthesis reached highest at the neonatal period (10–15 g/kg), which is five to seven times that of an adult (2 g/kg) (29). In contrast, P2 with *DUOXA1* p.R56W mutation, occurring at a highly conserved position among different species and resulting in DUOX1 protein being hampered by the failure to reconstitute active DUOX enzymes, manifested with permanent CH. Although the amount of DUOX2 expression was about five times more than DUOX1 and the DUOX2/DUOXA2 system was the primary source of H_2_O_2_ generation, the p.R56W mutant could cause a serious deficiency of thyroid hormone synthesis and eventually permanent CH. Our study indicated that P2 with permanent CH can be caused by monoallelic heterozygous missense *DUOXA1* mutation, which was consistent with previous results showing that biallelic (9), and possibly also monoallelic (30), *DUOXA2* mutations could cause permanent CH with partial iodide organification defect.

H_2_O_2_ generation experiment showed that p.R1307Q near the FAD binding domain impaired DUOX1 enzyme activity, while cycloheximide (CHX) chase experiment showed that the stability of DUOX1 protein was not changed based on the DUOX1 protein level at half-time (the half-life is 6 h predicted by ProtScale), which was consistent with the stability prediction by ProtScale, a bio-software which predicted that p.R1307Q mutant could not change the DUOX1 protein stability. After transfection with the *DUOXA1* expression vector, the group transfected with both p.R1307Q and p.R56W expression vector had the least amount of H_2_O_2_ generation. In contrast, the group transfected with both wild type *DUOX1* and wild type *DUOXA1* expression vectors had the largest amount of H_2_O_2_ generation. Moreover, the group with both wild type constructs had the maximum DUOX1 mRNA level and DUOX1 mRNA level decreased significantly in the group with p.R56W mutant vectors. As mentioned above, DUOXA is necessary for DUOX to form functional complexes to exert its biological function, hypothesis on the decreased *DUOX1* mRNA level caused by p.R56W *DUOXA1* mutation are as follows: (1) p.R56W *DUOXA1* mutation may participate in the transcriptional regulation, including transcriptional initiation and termination, and thus result in the decrease of *DUOX1*mRNA level. (2) p.R56W *DUOXA1* mutation may affect the *DUOX1* mRNA stability through destroy the structure of 3′ poly(A) tail and 3' UTR, and consequently cause the degradation of *DUOX1* mRNA. Consistently, the group with both WT expression vectors had the maximum DUOX1 protein expression and the group with both mutant expression vectors had the minimum DUOX1 protein expression. Functional studies indicated that the p.R1307Q mutant caused both partial functional loss of the DUOX1 activity and the protein expression. Moreover, introduction of the p.R56W mutant could impair the DUOX1 activity and the protein expression more profoundly, suggesting that DUOXA1 was necessary for DUOX1 to perform normal function. Consequently, these data, taken together, make it plausible to propose that the DUOX1/DUOXA1 system, when mutated, can cause CH.

In our study, patient 1 and patient 2 each only had one mutation even after screening on all exons of *DUOX1* (P1) and *DUOXA1* (P2), respectively. Based on the functional results in the present study and published data, the development of CH in the two patients could be mechanistically explained as follows: (1) Monoallelic *DUOX1/DUOXA1* mutations may cause CH just like *DUOX2* (31)/*DUOXA2* (30); (2) Intact DUOX1 is needed for full function of the H_2_O_2_ generating system and intact DUOXA1 is needed for maximal activity of DUOX1; (3) There may be concurrent genetic alterations such as mutations of relevant genes haven't been examined in the two patients. Future studies are needed to elucidate the specific molecular mechanisms involved.

In conclusion, we have identified two heterozygous missense mutations in *DUOX1* and *DUOXA1* in two Chinese patients with CH with goiter, respectively. Functional studies demonstrate that the two mutations each can partially impair the expression of DUOX1 and H_2_O_2_ generation and that intact DUOXA1 is important for the activity of DUOX1. Our study for the first time demonstrates that the DUOX1/DUOXA1 system, when genetically defective, can cause CH.

## Data Availability

The raw data supporting the conclusions of this manuscript will be made available by the authors, without undue reservation, to any qualified researcher.

## Ethics Statement

Our study was approved by the medical ethics committee of the Affiliated Hospital of Qingdao University and written informed consent was signed by each participant or their parents/legal guardians before they participated in the study.

## Author Contributions

SL and YG designed the study. WH analyzed the data and drafted the manuscript. WH, YZ, and HZ conducted the experiments. FW, HW, and XL collected the sample and analyzed the basic clinical data. SL, PJ, HW, YW, and XM interpreted the results and provided critical revisions of the manuscript. All authors approved the final version of the manuscript and agreed to be accountable for all aspects of the work.

### Conflict of Interest Statement

The authors declare that the research was conducted in the absence of any commercial or financial relationships that could be construed as a potential conflict of interest.

## Supplementary Material

Supplementary Figure 1 The standard calibration curve of H_2_O_2_ release and fluorescence intensity.

Supplementary Figure 2 The size of the bands of DUOX1 protein in western blot.
